# Summary of World Café Discussions Table 3: Dissemination

**DOI:** 10.25646/6509

**Published:** 2020-06-04

**Authors:** Thomas Ziese, Julia Truthmann

**Affiliations:** 1 Robert Koch Institute, Berlin, Department of Epidemiology and Health Monitoring; 2 Formerly Robert Koch Institute, Berlin, Department of Epidemiology and Health Monitoring

Translation and dissemination of scientific knowledge is essential to develop successful prevention strategies and involve all relevant stakeholders [1]. The question we answered was: ‘How can the Robert Koch Institute (RKI) as a national public health institute facilitate dissemination of results among public health stakeholders, and what could be important steps to enhance that (e.g. Cochrane Public Health Research network, institutional repositories)?’

First, we defined the stakeholders, which seem to be relevant for the dissemination of RKI research: among others Public Health Services (ÖGD), Health ministries (Federal, State), as well as policy areas (‘health in all policy’), media and press, universities, schools, the Federal Centre for Health Education (BZgA), the general public and health care providers.

Secondly, we discussed how the RKI can facilitate the dissemination of results among these stakeholders. Public health strategies comprise interventions at local, regional and national levels. These strategies should be organized in a conceptual framework. Thus, the RKI will benefit from the synergy effects resulting from actions on different levels. The exchange of experience, knowledge and ideas between the RKI and the stakeholders requires proactive as well as reactive structures. Proactive structures were defined as continuous working groups between the national and the federal state level to set priorities and present relevant heath issues. Reactive structures were considered as standing working groups (do not meet regularly, but members and responsibilities are predefined to enable fast workflows in case of urgent matters) between different stakeholders, which would enable a fast exchange of ideas and needs. To use windows of opportunity – time points where policy advice is required – flexibility and structures which enable fast responses are needed. Information preparedness could be realized through information repositories, which have to be updated on a regular basis. Furthermore, ad hoc working groups would be useful to work on current topics. Social media e.g. storytelling via Twitter seems to have a great potential to inform persons seeking for health information and patients about health issues [2].

Important steps to enhance dissemination include the need for a RKI mandate to ensure capacities for the implementation of strategies and permanent establishment of competencies. Secondly, goals have to be set, which include agenda setting: ‘What are the topics which need to be addressed? Do it actively.’ and agenda keeping: ‘What is in the public discussion? Influence it by facts.’ In addition, structures and networks have to be built up to increase expert knowledge and exchange. As discussed during the workshop, for instance, competencies of the RKI staff may be increased through exchange with World Health Organization (WHO) collaboration centers or a WHO collaboration center may be hosted at the RKI.
